# Correlation between ovarian morphology and biochemical and hormonal parameters in polycystic ovary syndrome

**DOI:** 10.12669/pjms.323.10082

**Published:** 2016

**Authors:** Cihan Inan, Cihan Karadag

**Affiliations:** 1 Dr. Cihan Inan, Department of Obstetrics and Gynecology, Kulu State Hospital, Konya, Turkey; 2 Dr. Cihan Karadag, Marmara University Pendik Training and Research Hospital, Department of Obstetrics and Gynecology, Istanbul, Turkey

**Keywords:** Hyperandrogenism, Insulin resistance, PCOS, PCO morphology, Total cholesterol

## Abstract

**Objective:**

To determine the biochemical and hormonal differences in polycystic ovary syndrome (PCOS) patients with and without polycystic ovary (PCO) morphology and to evaluate the outcomes resulting from those differences.

**Methods:**

The study included a total of 83 patients with PCOS; 43 of them had PCO morphology (Group-I) and 40 did not (Group-II). Serum LDL, HDL, total cholesterol, triglyceride (TG), total testosterone (T), follicle stimulating hormone (FSH), luteinizing hormone (LH), 17b-estradiol (E2), prolactin (PRL), thyroid stimulating hormone (TSH), sex hormone binding globulin (SHBG), glucose and insulin levels were determined. Homoeostatic model assessment insulin resistance (HOMA-IR) index was calculated.

**Results:**

The two groups were similar with respect to BMI. The systolic and diastolic blood pressure measurements of Group-I were significantly lower (p<0.01). Serum mean level of LH (p=0.026) and the mean LH/FSH (p=0.001) level of Group-I were significantly higher than Group-II. The total cholesterol and triglyceride levels of Group-I were significantly lower (p<0.05, p<0.01). The mean HOMA-IR level of Group-I was significantly lower than Group-II (p=0.004).

**Conclusions:**

The group without PCO morphology had a higher risk than the other group in terms of increased insulin resistance, dyslipidemia and cardiovascular diseases due to effects of hyperandrogenism.

## INTRODUCTION

Polycystic ovary syndrome (PCOS) is one of the most common endocrine disorder in reproductive aged women^1^ and characterized by oligo or anovulation, hyperandrogenism (clinical and/or biochemical) and presence of polycystic ovaries.^2^ The most common symptoms include hirsutism, irregular menstrual cycles, infertility problems, insulin resistance, dyslipidemia, hypertension, type-2 diabetes, coroner artery diseases and increased rates of metabolic syndrome.^3^,^4^

In the pathophysiology of PCOS, the pulsatile release of gonadotropic releasing hormone (GnRH) is disturbed, and this has a negative impact on the follicle development in ovaries. Some follicles do not fully mature, some follicles go into atresia, and some stay in the ovarian periphery in the form of small cysts.^5^ Polycystic-looking ovaries are not definite for diagnosing PCOS. Studies have reported considerably different rates in terms of the incidence of polycystic ovary (PCO) morphology in patients with PCOS^6^ and there are inconsistent results regarding the effects of PCO morphology in PCOS patients.

In this study, our purpose was to examine the differences between the possible biochemical and hormonal markers in the patients with PCOS with and without PCO morphology and to evaluate the outcomes resulting from those differences.

## METHODS

The study included a total of 83 patients that came to Kulu State Hospital between March 2013 and March 2014 and were diagnosed with PCOS. Forty three patients had PCO morphology (Group-I) and 40 patients did not (Group-II). Group-I was composed of patients who had PCO morphology and at least one of the other two criteria of PCOS (hyperandrogenism and/or oligomenorrhea-amenorrhea). Group-II was composed of patients who had hyperandrogenism (biochemical and/or clinical) and oligomenorrhea-amenorrhea without PCO morphology. Necessary ethical consent was received from the Ethics Council of Selcuk University School of Medicine. Informed consents were received from patients for the study. The study participants were in the age group of 18-40.

In 2003 Rotterdam criteria was used to diagnose PCOS: 1- oligomenorrhea-amenorrhea, 2- biochemical and/or clinical hyperandrogenism and 3- PCO morphology in ultrasonography (USG). Meeting at least two of those three criteria was the condition for PCOS diagnosis. The patients with suspicion of androgen-secreting tumor, hyperprolactinemia, Cushing syndrome, congenital adrenal hyperplasia were excluded in accordance with 2003 Rotterdam PCOS Consensus Workshop Group.^2^ Patients who had received medical treatment within the last 6 months and patients with clinical or biochemical features of thyroid dysfunction, diabetes mellitus were also excluded from the study.

USG examinations were performed on patients in the early follicular phase of menstrual cycle by using 6-8 MHz B mode pelvic and endovaginal probe (Mindray DC-T6- Shenzhen, China). Having 12 or more follicles of 2-9 mm even in one ovary and/or detecting an ovary volume over 10ml were accepted as PCO morphology.^2^,^7^ The body mass indexes of patients were calculated by using their ages, weights (kg) and heights (m) of patients (BMI=kg/m^2^). Modified Ferriman-Gallwey score (mFG) was evaluated for each participant. The patients with a mFG score over 8 were considered to have hirsutism. Systolic/diastolic blood pressures were measured after a ten minute rest. Peripheral blood samples were also taken from patients in the follicular phase of their menstrual cycles. Insulin resistance was calculated by using the formula: Homeostasis Model of Assessment–Insulin Resistance (HOMA-IR) = fasting glucose (mmol/L) x fasting Insulin (mU/mL) / 22.5.

Serum LDL, HDL, total cholesterol and triglyceride (TG) levels were measured by spectrophotometric method (Advia 1800, Siemens, Erlangen, Germany). Serum dehydroepiandrosterone sulfate (DHEAS) and sex hormone binding globulin (SHBG) levels were measured by chemiluminescence method (Immulite 2000, Siemens, Erlangen, Germany). Serum total testosterone (T), follicle stimulating hormone (FSH), luteinizing hormone (LH), 17b-estradiol (E2), prolactin (PRL), thyroid stimulating hormone (TSH) and insulin (Ins) levels were measured by immunoassay method (Advia Centaur XP, Siemens, Erlangen, Germany). Free testosterone (FT) and 17 hydroxyprogesterone (17OHPG) levels were measured by radioimmunoassay method (Immunoassays S.A, Diasource, Louvain-la-Neuve, Belgium). Serum glucose levels were determined by an enzymatic UV test (hexokinase method, AU5800, Beckman Coulter Inc., Brea, CA, USA).

NCSS (Number Cruncher Statistical System) 2007 (Kaysville, Utah, USA) program was used for statistical analysis. While evaluating study data, descriptive statistical methods were used. Student t-test was used for comparing two groups in terms of quantitative data with normal distribution, and Mann Whitney U test was used for comparing two groups in terms of data without normal distribution. Significance was evaluated at the levels of p<0.01 and p<0.05.

## RESULTS

The demographic parameters and laboratory results of study groups are shown in Table-I. The two groups were similar with respect to BMI. The mean age in PCOS group with PCO morphology was significantly lower (p<0.01). The systolic and diastolic blood pressure measurements of Group-I were significantly lower (p<0.01). Eighteen PCOS patients had hyperandrogenism (clinical and/or biochemical) in Group-I. All (40 patients) PCOS patients had hyperandrogenism (clinical and/or biochemical) in Group-II.

**Table-I:** Demographic parameters and laboratory results of study groups.

	*PCO morphology (+) (Group-I) (n=43)*	*PCO morphology (-) (Group-II) (n=40)*	*p*
Age (year)	23.42±5.31	27.65±5.59	0.001
BMI (kg/m^2^)	24.71±1.76	26.08±2.05	0.416
SBP(mm/Hg)	117.91±11.08	126.25±12.39	0.002
DBP(mm/Hg)	73.49±9.22	79.37±9.34	0.005
FSH (mIU/ml)	4.94±1.37	5.20±1.31	0.371
LH (mIU/ml)	8.43±2.43	7.20±2.48	0.026
LH/FSH	1.81±0.61	1.43±0.49	0.001
PRL (ng/ml)	13.19±6.24	12.43±3.66	0.733
TSH (uIU/ml)	1.71±1.16	1.89±0.67	0.039
E2 (pg/ml)	59.09±17.03	61.74±26.11	0.561
DHEAS (mcg/dl)	250.15±97.24	240.71±51.86	0.583
FT(pg/ml)	1.15±0.88	1.02±0.69	0.844
T (ng/ml)	0.59±0.15	0.57±0.12	0.084
SHBG(nmol/L)	23.68±8.24	26.41±1.21	0,056
Total cholesterol (mg/dl)	164.74±34.59	178.29±25.97	0.047
LDL (mg/dl)	104.03±26.60	107.20±21.05	0.553
HDL (mg/dl)	50.95±9.97	47.87±8.33	0.135
TG (mg/dl)	87.44±34.83	126.74±47.77	0.001
Glucose (mg/dl)	80.23±8.14	81.58±7.16	0.218
Ins (µU/ml)	10.71±3.14	13.55±4.19	0.001
HOMA- IR	2.12±1.04	2.73±0.66	0.004

BMI body mass index, SBP systolic blood pressure, DBP diastolic blood pressure, LH luteinizing hormone, FSH follicle stimulating hormone, E2 17b-estradiol, T total testosterone, FT free testosterone, DHEAS dehydroepiandrosterone sulfate, 17OH PG 17 hydroxyprogesterone, SHBG sex hormone binding globulin, PRL prolaktin, TSH thyroid stimulating hormone, Ins insuline, HOMA-IR Homeostatic Model Assessment–Insulin Resistance Index, TG trigliseride

Serum mean level of LH was significantly higher in Group-I (p=0.026). The LH/FSH levels of Group-I were higher compared to Group-II, and this was also statistically significant (p=0.001). Mean TSH level of Group-I was significantly lower (p<0.05).

While the LDL and HDL levels of patients had no statistically significant differences between the groups (p>0.05), the total cholesterol and triglyceride levels of Group-I were significantly lower (p<0.05, p<0.01). The HOMA-IR levels of Group-I were lower compared to Group-II, and this was also statistically significant (p=0.004).

## DISCUSSION

In this study we evaluated the two groups of PCOS patients who were composed of with and without PCO morphology. PCOS patients without PCO morphology had higher systolic/diastolic blood pressure measurements, higher total cholesterol, TG and HOMA-IR levels and lower LH and LH/FSH levels.

PCOS is a very heterogeneous disorder. Duijkers et al.^8^ indicated that PCO morphology can be seen in healthy women with regular menstrual cycles. Besides, it is known that ovary volume increases and cystic changes occur in primary hypothyroidism patients. Therefore, thyroid diseases should be excluded before making a diagnosis for PCOS. Muderris et al.^9^ studied 26 primary hypothyroidism patients and 26 healthy control patients and found that the hypothyroidism group had bigger ovary volumes. They reported that the cystic formation in ovary was not correlated with the TSH level. In our study, none of the patients had primary hypothyroidism. Compared to the other group, the group with PCO morphology had significantly lower TSH values. Although there was a TSH difference between groups, we think that this is not related to PCO morphology.

It is considered that there is a relation between PCOS and insulin resistance. We calculated the HOMA-IR levels to determine insulin resistance. The mean HOMA-IR level was higher in the group without PCO morphology. Since there was no significant difference between groups in terms of BMI, BMI has no effect on the difference between HOMA-IR levels. Moghetti et al.^10^ showed the association between hyperandrogenism and insulin resistance in women. The group without PCO morphology had higher number of hyperandrogenemic PCOS patients than the group which comprised of PCOS patients with PCO morphology. The possible effect of hyperandrogenism could cause increased insulin resistance in PCOS group without PCO.

Dyslipidemia is common among patients with PCOS. It is possible to have increased LDL, TG and decreased HDL along with PCOS.^11^ Dyslipidemia is related to unhealthy diets, obesity, metabolic syndrome, hyperandrogenism, physically inactive lifestyles, and genetic factors.^12^ In our study, although there was no significant difference between groups in terms of LDL and HDL, total cholesterol and TG levels were higher in the group without PCO morphology. Hyperandrogenism is considered a risk factor for dyslipidemia.^12^ There was no significant difference among our patients in two groups in terms of androgen levels. But the PCOS patients without PCO morphology were all diagnosed with clinical and/or biochemical hyperandrogenism. This high level of hyperandrogenic PCOS patients level could also cause high levels of total cholesterol and TG in PCOS patients without PCO morphology.

In our study, we found that the group with positive PCO morphology had an increased ratio of LH/FSH. Particularly this ratio might have negatively affected the follicle development in favor of LH. Parallel to the view accepted in literature, we think that this situation results in follicular atresia and the formation of follicles that cannot mature. Alsamarai et al.^13^ found that ovarian volume and follicle counts decreased with age in patients with PCOS. In their study, ovarian follicle count had a more rapid decrease with age compared to the control group that had regular menstrual cycle, and the decrease in ovarian volume was slower. In our study, the average age of the group with PCO morphology was lower compared to the other group. It is possible that the lower age might have had an effect on having a positive PCO morphology. We think that maybe the ovarian volume and follicle count will decrease as age increases in time, parallel to the literature. In our study the groups were BMI matched. This was the main limitation of our study. We could not assess the relationship between PCO morphology and BMI.

Hyperandrogenism and insulin resistance in PCOS stop the follicle development. The resulting follicle arrest contributes to PCO morphology where small antral follicles are lined in ovarian periphery.^14^ The change in the intra-ovarian paracrine signals has an important effect on the formation of PCO morphology. Particularly the emerging hyperandrogenic environment causes follicular arrest and prevents the formation of mature follicles. Despite that, in our study there was no difference between two groups in terms of free testosterone and DHEAS. It is possible that the sensitivity of androgen receptors in the ovary affects ovarian morphology as much as the androgen level. Although there was no difference between groups in terms of androgen levels, we think that, the possible differences in follicular androgen sensitivity might be the reason for seeing PCO morphology in a group of ovaries.

## CONCLUSION

The clinical and metabolic characteristics of each PCOS patient might present quite differently. In our study, we found statistically significant high levels of insulin resistance, triglyceride, total cholesterol, systolic and diastolic blood pressure values in PCOS group without PCO morphology, compared to PCOS group with PCO morphology. All patients without PCO morphology had hyperandrogenism and this outcomes may have resulted from hyperandrogenism’s effects. Therefore, we think that patients with PCOS without PCO morphology are under more risk in terms of metabolic disturbances such as increased risk of type-2 diabetes, coronary artery disease, hypercholesterolemia and thus metabolic syndrome due to effects of hyperandrogenism. Therefore, a more careful follow-up of patients with PCOS in this group can be considered with respect to the possibility of a future onset of cardiovascular diseases.
